# Strengthening Antimicrobial Stewardship in Nigeria: Qualitative and Quantitative Insights into Barriers, Enablers, and Implementation Strategies (2015–2025)

**DOI:** 10.1093/ofid/ofag476

**Published:** 2026-08-08

**Authors:** Omobolanle Margaret Abe, Rasha Abdelsalam Elshenawy

**Affiliations:** Department of Medicine, School of Health, Medicine and Life Sciences, University of Hertfordshire, Hatfield, UK; Department of Medicine, School of Health, Medicine and Life Sciences, University of Hertfordshire, Hatfield, UK

**Keywords:** antimicrobial resistance, antimicrobial stewardship, low- and middle-income countries, Nigeria, systematic review

## Abstract

**Background:**

Antimicrobial resistance is a critical global health threat. Nigeria faces particular challenges implementing antimicrobial stewardship (AMS) programs due to structural barriers and resource constraints. This review synthesizes evidence on barriers, facilitators, and strategies influencing AMS implementation in Nigerian healthcare facilities.

**Methods:**

A systematic search of 6 databases (PubMed, Scopus, Web of Science, the Cochrane Library, CINAHL, and Google Scholar; 1 January 2015–31 December 2025) identified English-language studies reporting AMS implementation in Nigerian healthcare settings. Quality was assessed using Critical Appraisal Skills Program and Mixed Methods Appraisal Tool. Data were synthesized using narrative and thematic analysis. Frequencies are expressed as the number (and percentage) of the 10 included studies reporting each item (denominator = 10 studies), not as proportions of pooled participants. The review was registered with PROSPERO (CRD420251165641) and followed PRISMA 2020 guidelines.

**Results:**

Ten studies (1343 participants) met the inclusion criteria. The most frequently reported barriers were insufficient training/knowledge (9/10, 90%), lack of guidelines and limited resources/funding (each 8/10, 80%), and poor surveillance systems (7/10, 70%). Leading facilitators were leadership support (8/10, 80%), multidisciplinary collaboration and education/training (each 7/10, 70%), and availability of guidelines (6/10, 60%). The most frequently reported implementation strategies were education and training (9/10, 90%), antibiotic-use audits and clinical-guideline development (each 8/10, 80%), and formulary restrictions/policies (7/10, 70%).

**Conclusions:**

This first Nigeria-specific systematic review identifies critical implementation gaps. Pharmacy-led, leadership-supported, and governance-embedded AMS programs show the greatest promise. Five evidence-based recommendations centered on policy reform, capacity building, diagnostic investment, AMS–Infection Prevention and Control integration, and sustainability planning are proposed.

## Teaser

This study provides qualitative and quantitative insights into the barriers and enablers influencing antimicrobial stewardship in Nigeria from 2015 to 2025. It highlights practical implementation strategies to strengthen stewardship across healthcare settings.

## Graphical Abstract

**Graphical Abstract:**
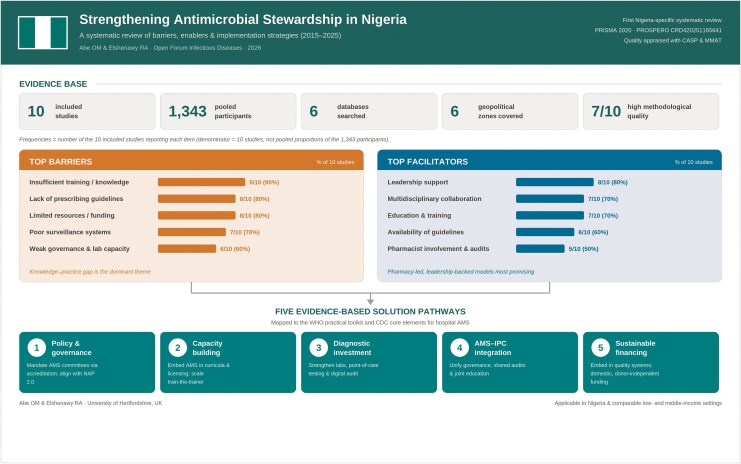

Antimicrobial resistance (AMR) is among the greatest threats to global health, directly responsible for over 1.2 million deaths in 2019 and projected to cause up to 10 million deaths annually by 2050 without decisive action [1, 2]. Nigeria, Africa's most populous nation, faces a critical AMR burden: antibiotic use among hospitalized patients is estimated at 65%–79% [3]. Yet formal antimicrobial stewardship (AMS) programs remain limited: a 2018 survey of tertiary hospitals found only 35% had a formal AMS team and 12% had audited antibiotic use [4].

Antimicrobial stewardship promotes responsible antimicrobial use to improve patient outcomes and reduce resistance [5]. Despite the launch of Nigeria's National Action Plan on AMR (NAP 1.0; 2017–2022) and the updated NAP 2.0 (2024–2028), implementation remains fragmented. Regulatory bodies, including the National Agency for Food and Drug Administration and Control (NAFDAC) and the Pharmacy Council of Nigeria (PCN), face inconsistent enforcement capacity [6]. While AMR demands a unified One Health response integrating human, animal, and environmental health [7], this review focuses on human healthcare settings, where the evidence base is concentrated and stewardship interventions are most urgently needed.

Key stakeholders include those in human health (healthcare professionals at all facility levels; community pharmacists), animal and environmental health (veterinarians and agricultural and environmental health officers), policymakers and regulators (the Nigeria Centre for Disease Control [NCDC], NAFDAC, PCN), and the public [8]. Despite their importance, barriers, facilitators, and strategies influencing AMS implementation in Nigerian healthcare have not been systematically synthesized [9]. This review addresses that gap, providing the first Nigeria-specific synthesis to inform policy and program development.

## The Recommended Components of AMS and the Nigerian Context

Internationally, the components of an effective hospital AMS program are well defined and provide the benchmark against which the barriers reported in this review can be interpreted. The United States Centers for Disease Control and Prevention describes 7 core elements—leadership commitment, accountability, pharmacy (drug) expertise, action (eg, facility-specific treatment guidelines, prospective audit and feedback, and preauthorization of selected agents), tracking of antibiotic use and resistance, reporting of these data to prescribers, and education [10]. For resource-limited settings, the World Health Organization (WHO) practical toolkit translates these into paired national- and facility-level core elements, emphasizing a national governance and coordination structure, a facility AMS committee and team, standard treatment guidelines aligned with the WHO Access, Watch, Reserve (AWaRe) classification, education and training, and surveillance of antimicrobial consumption and resistance [11, 12].

In Nigeria, this formal architecture is established in policy but unevenly operationalized. NAP 1.0 and NAP 2.0 set objectives spanning awareness, surveillance, infection prevention, optimized antimicrobial use, and sustainable investment, coordinated nationally by the NCDC through a multisectoral AMR Coordinating Committee and Technical Working Group, with antimicrobial regulation by NAFDAC and pharmacy-practice regulation by the PCN [6, 8]. Surveillance is being developed through enrollment in the WHO Global Antimicrobial Resistance and Use Surveillance System and national antimicrobial-consumption reporting, while national Standard Treatment Guidelines and the Essential Medicines List provide a prescribing reference [8]. At facility level, however, AMS committees, prescribing guidelines, audit and feedback, and laboratory-supported surveillance remain inconsistently implemented [4]. This gap between a comparatively well-developed national policy framework and limited facility-level execution is the central problem this review examines.

## METHODS

### Registration and Protocol

This review was registered with PROSPERO (CRD420251165641) prior to searching and followed PRISMA 2020 guidelines [13]. The PICO framework (Population: Nigerian healthcare facilities; Intervention: AMS programs or components; Comparison: not applicable; Outcome: barriers, facilitators, implementation strategies) structured the search. While PICO is primarily designed for comparative clinical questions, it was adapted here to frame a descriptive implementation question, a recognized approach in implementation science systematic reviews (Supplementary Table 1). Since this review analyzed existing published data, formal ethics approval was not required.

### Eligibility Criteria

Studies were included if (1) published in English; (2) conducted in any Nigerian healthcare facility (tertiary, secondary, primary, community pharmacy; public or private); (3) reporting on AMS implementation, evaluation, or description; (4) peer-reviewed qualitative, quantitative, or mixed-methods studies; and (5) published between 1 January 2015 and 31 December 2025. Commentaries, editorials, conference abstracts, and studies not conducted in healthcare settings were excluded.

### Search Strategy

Six databases were searched: PubMed, Scopus, Web of Science, the Cochrane Library, CINAHL, and Google Scholar. Boolean operators combined terms for AMS (including both “antimicrobial” and “antibiotic” variants), implementation, barriers, facilitators, healthcare facilities, and Nigeria (Supplementary Table 2). Duplicates were removed first, followed by title/abstract screening, then full-text review.

### Data Extraction and Quality Assessment

Of 76 articles retrieved for full-text review, 20 were inaccessible, leaving 56 assessed for eligibility. Data extraction was performed independently by 2 reviewers using a structured Microsoft Excel form (Supplementary Table 3) to capture study design, setting, population, barriers, facilitators, strategies, and outcomes. Study quality was assessed using Critical Appraisal Skills Program checklists (qualitative/observational studies) and Mixed Methods Appraisal Tool (mixed-methods studies). Higher-quality studies were given greater prominence in thematic interpretation.

### Data Synthesis

Narrative synthesis and thematic analysis were applied given heterogeneous study designs. Barriers and facilitators were coded into themes (infrastructural, human resource, behavioral, and policy related). Strategies were mapped against WHO core elements for hospital AMS programs.

Because the primary studies used heterogeneous terminology and metrics, 2 reviewers independently mapped each reported barrier, facilitator, and implementation strategy to a standardized set of operational categories (defined in Table 2), derived inductively from the extracted data and aligned with the WHO core elements [11]. Each category was counted once per study when reported, whether qualitatively (eg, in interview findings) or quantitatively (eg, as a survey proportion); the metric used by the original study did not affect counting. All frequencies are therefore expressed as the number, and percentage, of the 10 included studies reporting each category (denominator = 10 studies) and do not represent pooled proportions of the 1343 participants. Where a study did not report a given category, it was recorded as “not reported”, so the denominator remained 10 for every category. Disagreements were resolved by discussion.

## RESULTS

### Study Selection

**Figure 1.**
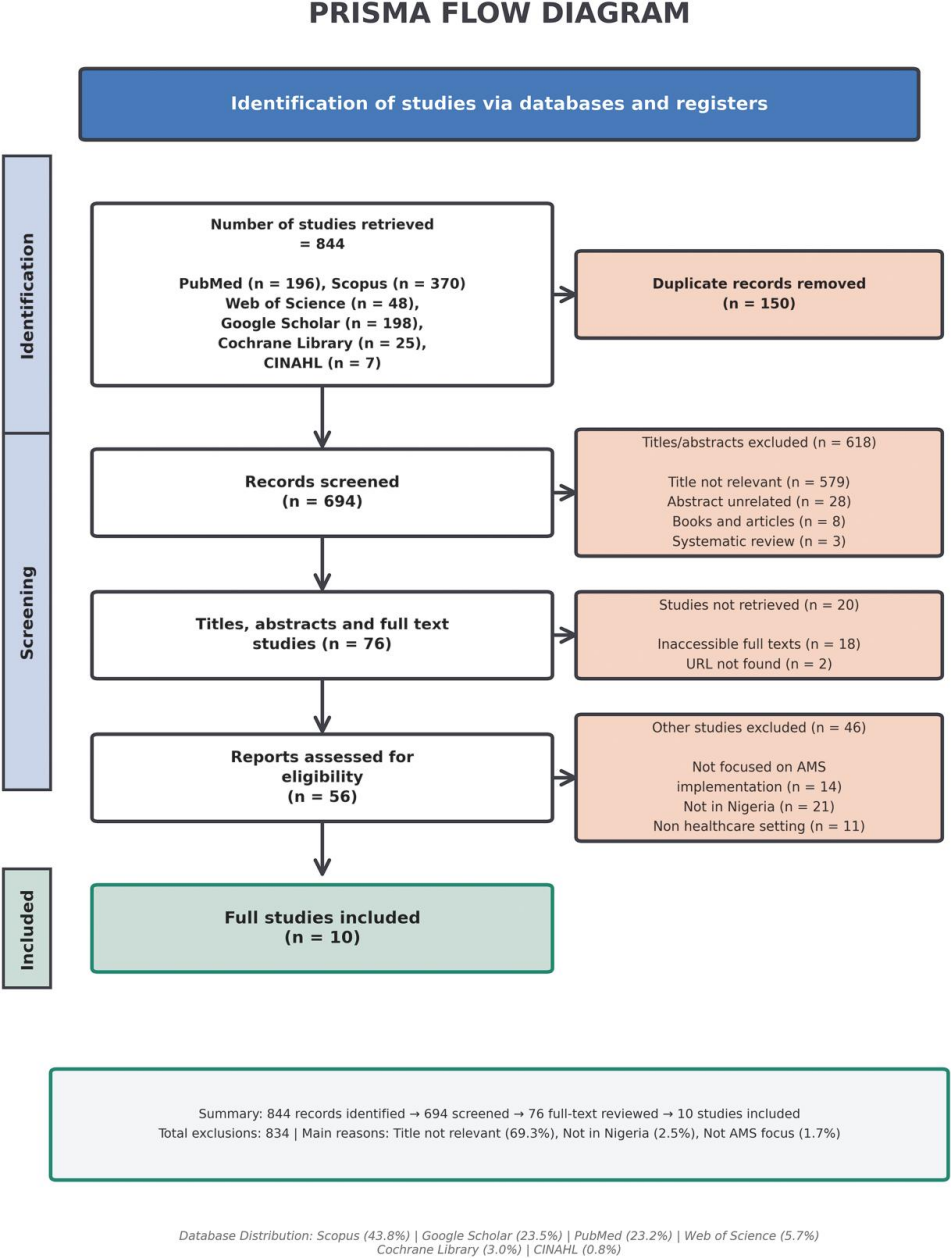
PRISMA 2020 flow diagram of study selection for the systematic review of antimicrobial stewardship implementation in Nigeria (search period: 1 January 2015–31 December 2025). Adapted from PRISMA 2020 statement (Page et al, 2021; retrieved 5/8/2025).

From 844 identified records (PubMed: 196; Scopus: 370; Web of Science: 48; Cochrane Library: 25; CINAHL: 7; Google Scholar: 198), 150 duplicates were removed, leaving 694 records for title/abstract screening; 618 were excluded at this stage. Of 76 retrieved for full-text review, 20 were inaccessible, leaving 56 for eligibility assessment. Forty-six were excluded (not AMS implementation focused: n = 14; not Nigeria: n = 21; not healthcare setting: n = 11). Ten studies met the inclusion criteria (Figure 1).

### Characteristics of Included Studies

Ten studies (2015–2025) encompassed 1343 participants across Nigerian healthcare facilities, representing all 6 geopolitical zones; Lagos State contributed 30% of the studies, and several adopted a nationwide scope (Table 1). Tertiary hospitals (40%) and mixed-level facilities (40%) predominated. Cross-sectional surveys were most common (40%), followed by qualitative interview studies (20%). Pharmacists featured in 60% of studies. Quality assessment showed 70% (7/10) high and 30% (3/10) moderate methodological quality (Table 1). The unit of sampling varied across studies—comprising healthcare facilities (eg, 25 facilities), healthcare workers (eg, 417 and 37 respondents), hospitalized patients (eg, 450 outpatient records and 260 pediatric inpatients), and community pharmacists (98)—so the pooled total of 1343 reflects heterogeneous sampling units rather than a single participant population. Publication peaks in 2021 and 2024 reflect growing recognition of AMS as a public health priority.

**Table 1.** Characteristics of the 10 Included Studies

Study (y)	Setting and Facility Level	Design	Population (Sampling Unit)	n	Quality
Chukwu et al. (2021)	Lagos; tertiary, secondary, primary, and private	Descriptive cross-sectional survey	Healthcare facilities	25	Moderate
Akpan et al. (2024)	Akwa Ibom; primary and secondary	Cross-sectional survey	Healthcare workers	417	Moderate
Oli et al. (2021)	Tertiary institution	Retrospective cross-sectional	Outpatient prescriptions/pharmacists	450	High
Kpokiri et al. (2020)	Bayelsa (Niger Delta); tertiary	Mixed-methods	Physicians	17	High
Chukwu et al. (2024)	Lagos; secondary and tertiary	Qualitative interviews	Doctors and pharmacists	25	High
Akitan et al. (2024)	Lagos; tertiary	Cross-sectional point-prevalence survey	Pediatric inpatients	260	High
Abubakar and Tangiisuran (2020)	Nationwide; tertiary	Electronic cross-sectional survey	Pharmacy/ID/AM pharmacists	37	High
Kamere et al. (2022)	Eight African countries including Nigeria	Scoping review	National AMS programs	N/A	High
Aika et al. (2022)	Benin City, Edo; primary, secondary, and tertiary	Cross-sectional qualitative interview	Healthcare managers	14	High
Abubakar (2020)	Minna and Kaduna; community pharmacies	Cross-sectional survey	Community pharmacists	98	Moderate

The sampling unit differs across studies (facilities, workers, patients, or pharmacists); the pooled total of 1343 therefore reflects heterogeneous units. Full extraction is provided in Supplementary Table 3.

Abbreviations: AM, antimicrobial; AMS, antimicrobial stewardship; ID, infectious diseases.

### Barriers and Facilitators

**Figure 2.**
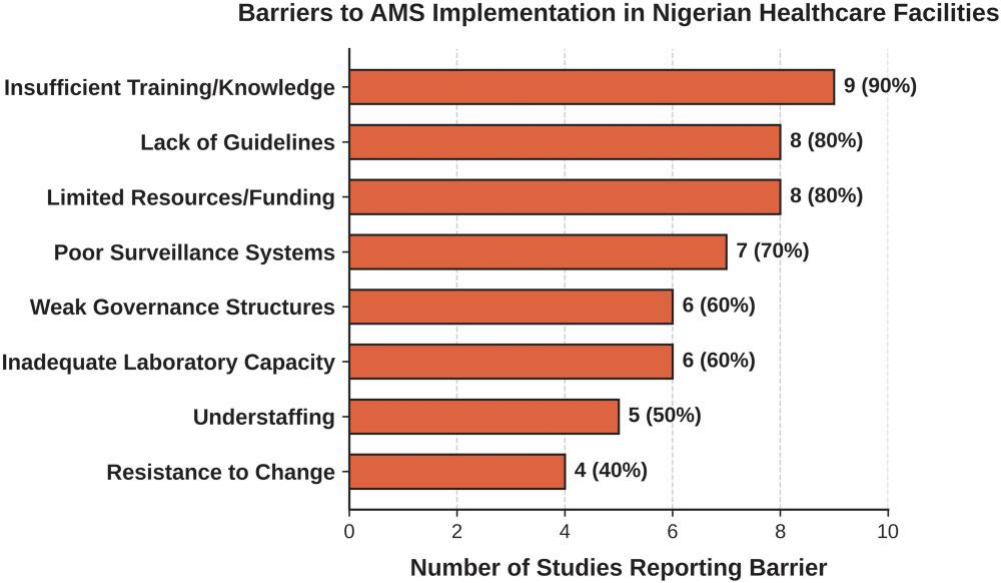
Barriers to antimicrobial stewardship (AMS) implementation in Nigerian healthcare facilities, presented in descending order of frequency (number and percentage of the 10 included studies reporting each barrier).

Frequencies below denote the number of the 10 included studies reporting each item. Across the 10 studies, insufficient training and knowledge was the most frequently reported barrier, identified in 9 studies (90%). This was followed by lack of, or inadequate, prescribing guidelines and limited resources/funding, each reported in 8 studies (80%); poor surveillance systems in 7 studies (70%); weak governance structures and inadequate laboratory capacity, each in 6 studies (60%); understaffing in 5 studies (50%); and prescriber resistance to change in 4 studies (40%) (Figure 2).

**Figure 3.**
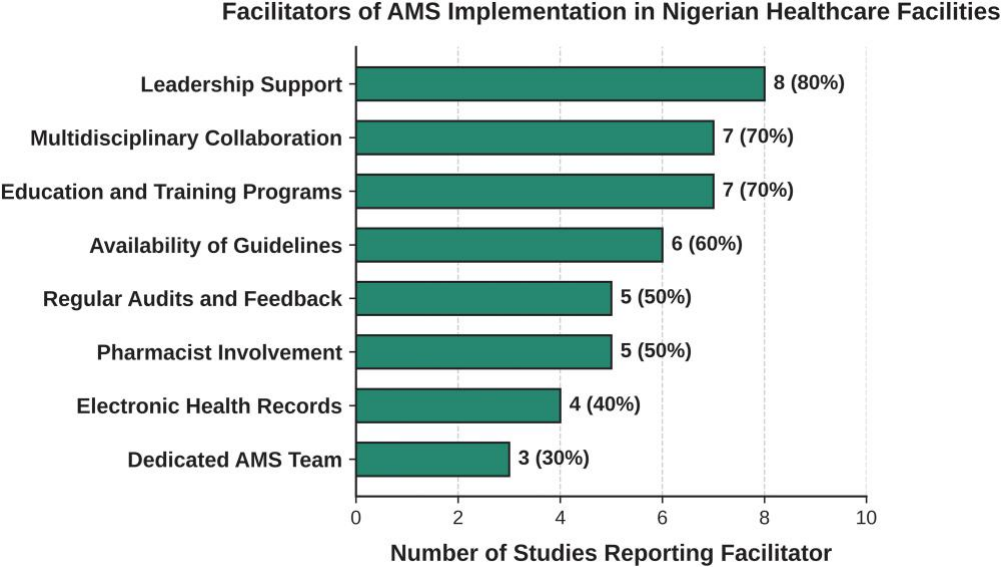
Facilitators of antimicrobial stewardship (AMS) implementation in Nigerian healthcare facilities, presented in descending order of frequency (number and percentage of the 10 included studies reporting each facilitator).

On the facilitator side, leadership support was the most frequently reported enabler, present in 8 studies (80%), followed by multidisciplinary collaboration and education/training programs, each in 7 studies (70%); availability of prescribing guidelines in 6 studies (60%); regular audit and feedback and pharmacist involvement, each in 5 studies (50%); electronic health records in 4 studies (40%); and a dedicated AMS team in 3 studies (30%; Figure 3). Notably, prescribing guidelines functioned as a barrier where absent or inadequate (8/10, 80%) and as a facilitator where available (6/10, 60%), reflecting heterogeneity across facilities.

### Implementation Strategies

**Figure 4.**
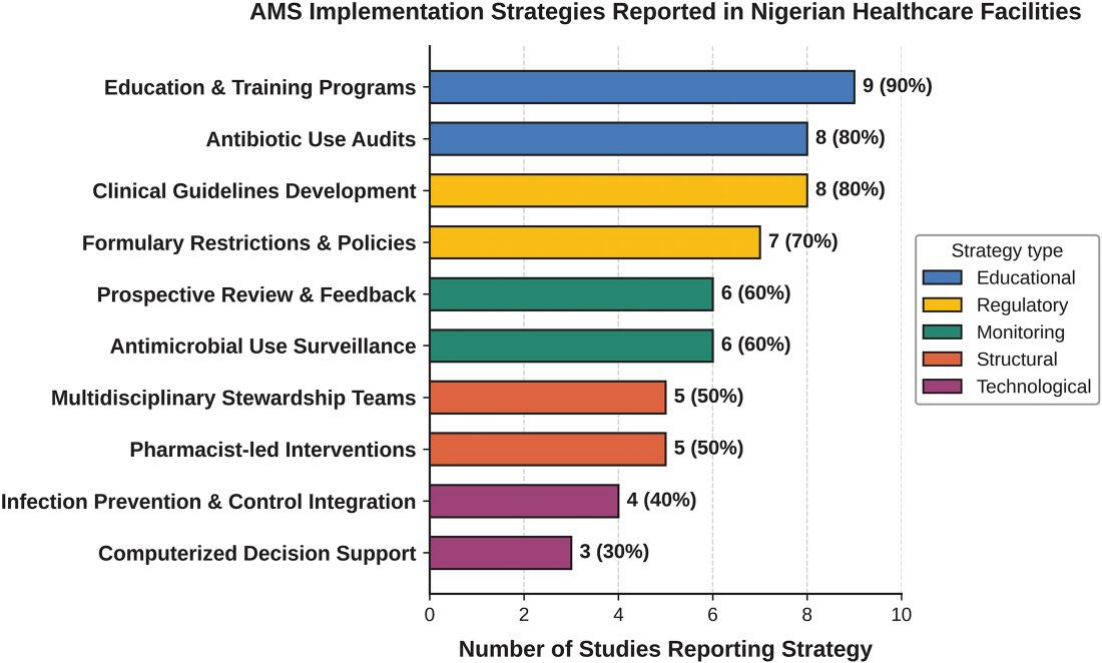
Antimicrobial stewardship (AMS) implementation strategies reported in Nigerian healthcare facilities, by frequency of reporting across the 10 included studies.

Education and training was the most commonly reported implementation strategy, used in 9 studies (90%), followed by antibiotic-use audits and clinical-guideline development, each in 8 studies (80%); formulary restrictions and policies in 7 studies (70%); prospective review and feedback and antimicrobial-use surveillance, each in 6 studies (60%); multidisciplinary stewardship teams and pharmacist-led interventions, each in 5 studies (50%); infection prevention and control (IPC) integration in 4 studies (40%); and computerized decision support in 3 studies (30%; Figure 4). Grouped by type, Nigerian facilities most often deployed educational and regulatory strategies, with technological solutions least common.

### Qualitative Thematic Analysis

#### Theme 1: Knowledge–Practice Gap

Deficits in formal preservice AMS education were reported in 70% of studies, whilst 90% reported insufficient in-service training, representing distinct dimensions: the 70% reflects prequalification curriculum gaps, while the 90% captures continuing professional development deficits. Where structured, pharmacist-led training was available, improved prescribing behavior and AWaRe classification adherence were observed.

#### Theme 2: Organizational Readiness and Leadership

Facilities with AMS committees and administrative backing demonstrated more structured stewardship and regular prescriber feedback. Absent institutional support produced fragmented, donor-dependent programs with limited long-term viability. Incorporating AMS performance metrics into accreditation frameworks emerged as a practical mechanism for institutionalizing leadership commitment.

#### Theme 3: Resource Constraints and System Fragmentation

Fragmented governance operated at 3 levels simultaneously: laboratory (absent antibiogram reporting), facility (absent AMS committees and accountability structures), and national (inconsistent policy enforcement and weak cross-sector coordination). These structural barriers compelled empirical prescribing and undermined surveillance. The absence of IPC integration emerged as a key structural gap, consistent with global evidence that AMS and IPC are most effective when unified.

#### Theme 4: The Pharmacist Role in AMS

Pharmacists were identified as key facilitators in 50% of studies (Figures 3 and 4), leading prescription reviews, audit and feedback, and in-service education. While not captured as a standalone barrier in Figure 2, their systematic underutilization represents an implicit system-level barrier—an opportunity cost functioning as a structural impediment to stewardship capacity.

### Integration of Findings

**Figure 5.**
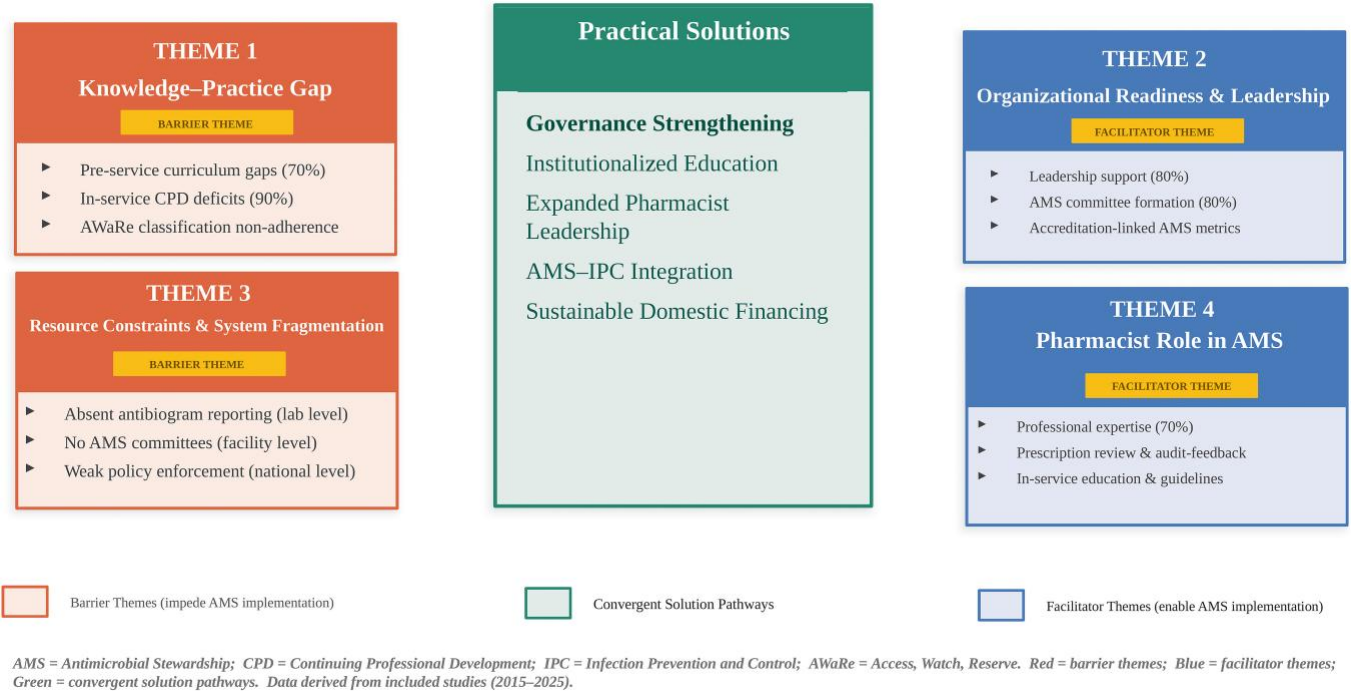
Integrated conceptual framework synthesizing barriers and facilitators to antimicrobial stewardship (AMS) implementation in Nigerian healthcare facilities (2015–2025).

Quantitative data (Figures 2–4) documented the frequency of barriers, facilitators, and strategies; qualitative data explained the underlying contextual dynamics. Figure 5 integrates these findings into a conceptual framework comprising 4 themes. Two operate predominantly as barriers—the knowledge–practice gap (Theme 1) and resource constraints with system fragmentation (Theme 3)—and 2 predominantly as facilitators—organizational readiness and leadership (Theme 2) and the pharmacist role in AMS (Theme 4). The framework converges on 5 solution pathways: governance strengthening, institutionalized education, expanded pharmacist leadership, AMS–IPC integration, and sustainable domestic financing (Figure 5).

## DISCUSSION

This systematic review synthesizes evidence on barriers, facilitators, and implementation strategies for AMS in Nigerian healthcare facilities, revealing a persistent gap between national policy ambitions and facility-level practice driven by structural, governance, and workforce constraints. These findings are consistent with global and regional literature demonstrating that sustainable stewardship in low- and middle-income countries (LMICs) requires context-adapted models supported by diagnostic strengthening, workforce investment, and phased implementation [14, 15]. A 2026 qualitative study of Nigeria's private healthcare sector reinforced this picture, identifying weak regulation, limited diagnostics, and workforce shortages as principal barriers, whilst highlighting training, partnerships, and private-sector engagement as key facilitators [9].

Placing Nigeria's experience alongside other African countries clarifies both shared and distinctive challenges. South Africa offers the most mature example: a pharmacist-driven, prospective audit and feedback program implemented across 47 private hospitals with limited infectious-disease expertise achieved an ∼18% reduction in antibiotic consumption (measured as defined daily doses), demonstrating that centrally coordinated, leadership-endorsed, pharmacist-led stewardship can be scaled even where specialist resources are scarce [16]. Across the continent, a systematic review of African AMS programs found that, although most countries had national action plans, few had translated them into adopted facility-level AMS guidelines—Kenya being a notable exception—with surveillance, funding, and One Health integration remaining the principal implementation gaps [14]. Nigeria's barriers are therefore largely shared with its regional peers (weak surveillance, constrained diagnostics, and funding dependence), but its specific configuration—a populous, federated health system with a large, weakly regulated private and retail-pharmacy sector—heightens the importance of the enablers identified here: visible leadership, multidisciplinary AMS committees, and expanded pharmacist roles, which underpin South Africa's comparatively successful model [14, 16].

Structural constraints, particularly under-resourced laboratory systems and poor diagnostic infrastructure, were among the most consistently reported barriers, aligning with African and global reviews that document limited AMS gains despite demonstrable improvements in guideline adherence, prescribing appropriateness, antibiotic use, and cost reduction [14, 15]. These system-level deficits are compounded by Nigeria-specific pressures: erratic medicine supply chains, high patient demand for antibiotics, and profit-driven prescribing in private facilities [9]. Community-level stewardship faces additional challenges, as a 2026 sub-Saharan Africa retail-medicine review found median nonprescription antibiotic dispensing of 67.5%, driven by weak regulation, economic pressures, and persistent patient demand despite AMR awareness [17]. The COVID-19 pandemic further exacerbated these vulnerabilities by increasing empirical antibiotic use and diverting already limited diagnostic resources [18, 19].

On the facilitator side, leadership commitment and AMS committee involvement were the most consistently reported enablers, consistent with international evidence that visible institutional leadership is foundational for cultural change and stewardship sustainability. This is reinforced by a 2023 Nigerian study conducted across 2 public healthcare institutions (n = 160), which found that 69% of surveyed healthcare professionals believed AMS could be implemented and sustained, with training and uniform antibiotic guidelines identified as key enabling factors [20]; the settings, sampling units, and sample sizes of all included studies are summarized for comparison in Table 1. Where pharmacist-led interventions—including prescription review, audit–feedback, and in-service education—were implemented, facilities reported improved adherence to prescribing guidelines, reinforcing the global evidence base for pharmacist-led AMS in resource-limited settings [21, 22].

### Strengths and Limitations

Strengths include: the first systematic review to focus exclusively on Nigeria; inclusion of diverse study designs, enabling evidence triangulation; and coverage of both hospital and community pharmacy settings. Limitations include: a small evidence base (n = 10); predominance of urban and tertiary settings limiting rural generalizability; potential publication bias toward positive outcomes; IPC not included as a standalone search term; heterogeneous terminology and metrics across primary studies, which required reviewer-led harmonization of reported items into standardized categories (Table 2); and a human health focus that does not fully capture One Health dimensions, an important area for future research.

**Table 2.** Frequency of Reported Barriers, Facilitators, and Implementation Strategies Across the 10 Included Studies, With Operational Definitions (Denominator = 10 Studies)

Category	n	%	Operational Definition (basis for Counting a Study)
Barriers			
Insufficient training/knowledge	9	90	Study reports inadequate preservice and/or in-service AMS education or low AMS/AWaRe awareness.
Lack of, or inadequate, guidelines	8	80	Study reports absent, outdated, or nonadopted prescribing/AMS guidelines.
Limited resources/funding	8	80	Study reports financial, staffing, or material constraints limiting AMS activity.
Poor surveillance systems	7	70	Study reports absent or weak antimicrobial use and/or resistance surveillance.
Weak governance structures	6	60	Study reports absent AMS committees, accountability, or policy enforcement.
Inadequate laboratory capacity	6	60	Study reports limited microbiology/diagnostic or antibiogram capacity.
Understaffing/workload	5	50	Study reports staff shortages or workload pressure impeding stewardship.
Resistance to change	4	40	Study reports prescriber or cultural resistance to stewardship practice.
Facilitators			
Leadership support	8	80	Study reports management/administrative backing for AMS.
Multidisciplinary collaboration	7	70	Study reports team-based, cross-professional stewardship working.
Education and training programs	7	70	Study reports structured AMS training as an enabler.
Availability of guidelines	6	60	Study reports presence/use of prescribing or AMS guidelines.
Regular audit and feedback	5	50	Study reports prospective/retrospective audit with prescriber feedback.
Pharmacist involvement	5	50	Study reports active pharmacist participation in AMS.
Electronic health records/IT	4	40	Study reports IT/EHR support for stewardship.
Dedicated AMS team	3	30	Study reports a formally constituted AMS team.
Implementation strategies			
Education and training	9	90	Study describes educational interventions for prescribers/staff.
Antibiotic-use audits	8	80	Study describes auditing of antibiotic prescribing/consumption.
Clinical-guideline development	8	80	Study describes development/adoption of prescribing guidelines.
Formulary restrictions and policies	7	70	Study describes formulary control or preauthorization policies.
Prospective review and feedback	6	60	Study describes prospective audit and feedback workflows.
Antimicrobial-use surveillance	6	60	Study describes monitoring of antimicrobial consumption/use.
Multidisciplinary stewardship teams	5	50	Study describes establishing AMS teams/committees.
Pharmacist-led interventions	5	50	Study describes pharmacist-delivered stewardship activities.
IPC integration	4	40	Study describes linking AMS with infection prevention and control.
Computerized decision support	3	30	Study describes digital decision-support or e-prescribing tools.

n and % refer to the number and proportion of the 10 included studies reporting each category. A category not reported by a study was coded “not reported”; the denominator remained 10 for every category.

Abbreviations: AMS, antimicrobial stewardship; AWaRe, Access/Watch/Reserve; EHR, electronic health record; IPC, infection prevention and control; IT, information technology.

### Actionable Recommendations

Five actionable recommendations provide a clear, implementation-focused pathway for strengthening AMS within Nigeria's health system, aligning with established national and global frameworks that emphasize governance, workforce development, diagnostics, integration, and sustainability as core pillars of effective stewardship [23–27] (Table 3). First, policy reform should mandate AMS committees as a condition of hospital accreditation, alongside antibiotic prescribing reporting frameworks and defined performance benchmarks, with state health ministry holding dedicated oversight and budget lines aligned with NAP 2.0. Second, capacity building must move beyond one-off training by embedding AMS competencies into undergraduate curricula and professional licensing, supported by scalable train-the-trainer and AMS champion models. Third, diagnostic investment is critical, encompassing strengthened microbiology laboratories, expanded point-of-care testing, and digital prescription monitoring for real-time audit and feedback. Fourth, integrating AMS with IPC through unified governance, shared audits, and combined education aligns with WHO recommendations and optimizes limited resources. Finally, sustainability planning should embed AMS within hospital quality improvement systems and national health information platforms, with co-leadership across professional bodies and reduced reliance on donors to ensure long-term continuity and health system resilience.

**Table 3.** Evidence-based Recommendations for Strengthening Antimicrobial Stewardship in Nigeria

Domain and Recommendation	Key Actions
1. Policy reform (governance)	Mandate AMS committees as a condition of hospital accreditation; introduce antibiotic-prescribing reporting frameworks; define measurable performance benchmarks; align state health ministries with NAP 2.0 governance; establish dedicated AMS budget lines.
2. Capacity building (workforce)	Embed AMS in undergraduate medical and pharmacy curricula; integrate into professional licensing examinations; implement train-the-trainer models; establish AMS champion networks; support scalable, cost-effective pathways.
3. Diagnostic investment (diagnostics)	Strengthen microbiology laboratories with antibiogram reporting; expand point-of-care diagnostics in primary care; implement digital prescription monitoring; enable real-time audit and feedback; and embed financing in hospital budgets (not donor dependent).
4. AMS–IPC integration (integration)	Adopt unified AMS–IPC governance structures; establish shared committees and combined audits; deliver integrated education programs; align with WHO hospital stewardship guidance; and optimize limited human resources.
5. Sustainability planning (sustainability)	Embed AMS in hospital quality-improvement frameworks; integrate with national health information systems; promote co-leadership across professional bodies; reduce dependency on donor funding; and ensure long-term health-system resilience.

Recommendations derived from synthesis of the 10 included studies (2015–2025) and alignment with NAP 2.0 and WHO stewardship frameworks. Relocated from former Supplementary Table 2.

Abbreviations: AMS, antimicrobial stewardship; IPC, infection prevention and control; NAP, National Action Plan; WHO, World Health Organization.

### Conclusions

This review identifies the barriers, facilitators, and strategies shaping AMS implementation in Nigeria. Insufficient training, lack of prescribing guidelines, and limited resources are the principal barriers; leadership commitment, multidisciplinary collaboration, professional expertise, and pharmacist engagement are the primary facilitators. Education and training, antibiotic-use audits, and clinical-guideline development are the most commonly reported strategies. The knowledge–practice gap is the dominant theme. Despite strong human resource potential, structural barriers constrain sustainability. The 5 evidence-based recommendations provide actionable guidance for Nigeria and comparable LMIC settings. Future research should target rural and private-sector facilities, evaluate cost-effectiveness, and explore AMS–IPC integration, ensuring stewardship is institutionalized through policy enforcement, stable financing, and professional education.

## Supplementary Material

ofag476_Supplementary_Data

## Supplementary Data

Supplementary materials are available at *Open Forum Infectious Diseases* online. Consisting of data provided by the authors to benefit the reader, the posted materials are not copyedited and are the sole responsibility of the authors, so questions or comments should be addressed to the corresponding author.
