# Supplementary material for: Strengthening Antimicrobial Stewardship in Nigeria: Qualitative and Quantitative Insights into Barriers, Enablers, and Implementation Strategies (2015–2025)
Source: Open Forum Infect Dis. 2026 Aug 8;13(8):ofag476. doi: 10.1093/ofid/ofag476 (PMC13469695; doi:10.1093/ofid/ofag476)
Supplement: Supplementary file 1 [file ofag476_supplementary_data.docx]

**Supplementary Materials**

**Strengthening Antimicrobial Stewardship in Nigeria: Qualitative and Quantitative Insights into Barriers, Enablers, and Implementation Strategies (2015–2025)**

Omobolanle Margaret Abe, Rasha Abdelsalam Elshenawy

Open Forum Infectious Diseases · Manuscript OFID-D-26-00473 · PROSPERO CRD420251165641

*This file contains Supplementary Tables S1–S3. Supplementary material is not copy-edited or typeset; its content is the responsibility of the authors.*

**Supplementary Table S1.** PICO framework and study eligibility criteria.

| **PICO element** | **Operationalisation in this review** |
| --- | --- |
| Population (P) | Patients and healthcare professionals in Nigerian healthcare facilities across all levels of care — tertiary, secondary, and primary hospitals and community pharmacies; public or private. |
| Intervention (I) | Antimicrobial stewardship (AMS) programmes or individual AMS components (e.g., facility prescribing guidelines, prospective audit and feedback, formulary restriction or pre-authorisation, education and training, and antimicrobial-use surveillance). |
| Comparison (C) | Not applicable. The review addresses a descriptive implementation question rather than a comparative effectiveness question. |
| Outcome (O) | Reported barriers to, facilitators of, and strategies for AMS implementation in Nigerian healthcare facilities. |

*PICO, Population/Intervention/Comparison/Outcome. PICO is primarily designed for comparative clinical questions; here it was adapted to frame a descriptive implementation question, a recognised approach in implementation-science systematic reviews.*

**Eligibility criteria**

| **Inclusion criteria** | **Exclusion criteria** |
| --- | --- |
| - Published in English - Conducted in any Nigerian healthcare facility (tertiary, secondary, or primary hospital; community pharmacy; public or private) - Reporting AMS implementation, evaluation, or description - Peer-reviewed qualitative, quantitative, or mixed-methods study - Published 1 January 2015 – 31 December 2025 | - Commentaries, editorials, or conference abstracts - Studies not conducted in a healthcare setting - Studies not conducted in Nigeria - Articles not focused on AMS implementation |

**Supplementary Table S2.** Search strategy: concept blocks, illustrative search terms, and database yields.

**(a) Concept blocks and search terms**

| **Concept** | **Search terms (combined with OR within each block)** |
| --- | --- |
| 1. Antimicrobial stewardship | “antimicrobial stewardship” OR “antibiotic stewardship” OR “antimicrobial steward*” OR “antibiotic steward*” OR AMS OR “antimicrobial use optimi*” OR “rational antibiotic use” OR “prudent antibiotic use” |
| 2. Implementation | implement* OR adopt* OR uptake OR establish* OR programme* OR program* OR practice OR delivery |
| 3. Barriers | barrier* OR challenge* OR obstacle* OR constraint* OR difficult* |
| 4. Facilitators | facilitat* OR enabler* OR driver* OR “enabling factor*” OR opportunit* |
| 5. Healthcare setting | hospital* OR “health* facilit*” OR clinic* OR “primary care” OR “secondary care” OR “tertiary care” OR pharmac* |
| 6. Nigeria | Nigeria* |

**Combined query:** (#1) AND (#6) AND (#2 OR #3 OR #4) AND (#5), limited to 2015–2025 and English language.

*Boolean operators, truncation symbols, and field tags were adapted to the syntax of each database. The exact field tags and final run date should be confirmed by the authors against the search log.*

**(b) Records identified and screened (PRISMA 2020)**

| **Stage / source** | **Records, n** |
| --- | --- |
| PubMed | 196 |
| Scopus | 370 |
| Web of Science | 48 |
| Cochrane Library | 25 |
| CINAHL | 7 |
| Google Scholar | 198 |
| **Total records identified** | **844** |
| Duplicates removed | 150 |
| Records screened (title/abstract) | 694 |
| Excluded at title/abstract screening | 618 |
| Reports sought for retrieval (full text) | 76 |
| Reports not retrieved / inaccessible | 20 |
| Reports assessed for eligibility | 56 |
| Excluded at full text (not AMS implementation 14; not Nigeria 21; not healthcare setting 11) | 46 |
| **Studies included in synthesis** | **10** |

*Search period 1 January 2015 – 31 December 2025. Counts correspond to the PRISMA 2020 flow diagram (Figure 1).*

**Supplementary Table S3.** Data extraction summary of the 10 included studies.

**(a) Study characteristics**

| **#** | **Study (year)** | **Setting & facility level** | **Design** | **Population (sampling unit)** | **n** | **Quality (tool; rating)** | **AMS focus / scope** |
| --- | --- | --- | --- | --- | --- | --- | --- |
| 1 | Chukwu et al. (2021) | Lagos; tertiary, secondary, primary & private | Descriptive cross-sectional survey | Healthcare facilities | 25 | CASP; Moderate | Facility-level assessment of AMS structures and implementation across mixed-level facilities. |
| 2 | Akpan et al. (2024) | Akwa Ibom; primary & secondary | Cross-sectional survey | Healthcare workers | 417 | CASP; Moderate | Survey of healthcare-worker knowledge, attitudes and practice relating to AMS. |
| 3 | Oli et al. (2021) | Tertiary institution | Retrospective cross-sectional | Outpatient prescriptions / pharmacists | 450 | CASP; High | Retrospective review of outpatient antibiotic prescribing. |
| 4 | Kpokiri et al. (2020) | Bayelsa (Niger Delta); tertiary | Mixed-methods | Physicians | 17 | MMAT; High | Development and implementation of an AMS programme in a tertiary hospital. |
| 5 | Chukwu et al. (2024) | Lagos; secondary & tertiary | Qualitative interviews | Doctors & pharmacists | 25 | CASP; High | Qualitative exploration of AMS barriers and enablers among prescribers and pharmacists. |
| 6 | Akitan et al. (2024) | Lagos; tertiary | Cross-sectional point-prevalence survey | Paediatric inpatients | 260 | CASP; High | Point-prevalence survey of paediatric antibiotic use. |
| 7 | Abubakar & Tangiisuran (2020) | Nationwide; tertiary | Electronic cross-sectional survey | Pharmacy / ID / AM pharmacists | 37 | CASP; High | National survey of AMS practice among hospital pharmacists. |
| 8 | Kamere et al. (2022) | Eight African countries incl. Nigeria | Scoping review | National AMS programmes | N/A | CASP; High | Scoping review of national AMS programmes (Nigeria among eight countries). |
| 9 | Aika et al. (2022) | Benin City, Edo; primary, secondary & tertiary | Cross-sectional qualitative interview | Healthcare managers | 14 | CASP; High | Qualitative interview study of AMS implementation with healthcare managers. |
| 10 | Abubakar (2020) | Minna & Kaduna; community pharmacies | Cross-sectional survey | Community pharmacists | 98 | CASP; Moderate | Survey of antibiotic dispensing and AMS practice among community pharmacists. |

*AM, antimicrobial; AMS, antimicrobial stewardship; CASP, Critical Appraisal Skills Programme; ID, infectious diseases; MMAT, Mixed Methods Appraisal Tool. CASP was applied to qualitative/observational studies and MMAT to mixed-methods studies. Methodological quality: 7/10 (70%) high, 3/10 (30%) moderate. The pooled total of 1,343 participants reflects heterogeneous sampling units.*

**(b) Barriers, facilitators, and implementation strategies reported per study**

| **#** | **Study (year)** | **Barriers reported** | **Facilitators reported** | **Implementation strategies reported** | **Main findings / outcomes** |
| --- | --- | --- | --- | --- | --- |
| 1 | Chukwu et al. (2021) | *[map to S-Table 2 barrier categories]* | *[map to S-Table 2 facilitator categories]* | *[map to S-Table 2 strategy categories]* | *[enter key finding/outcome from extraction]* |
| 2 | Akpan et al. (2024) | *[map to S-Table 2 barrier categories]* | *[map to S-Table 2 facilitator categories]* | *[map to S-Table 2 strategy categories]* | *[enter key finding/outcome from extraction]* |
| 3 | Oli et al. (2021) | *[map to S-Table 2 barrier categories]* | *[map to S-Table 2 facilitator categories]* | *[map to S-Table 2 strategy categories]* | *[enter key finding/outcome from extraction]* |
| 4 | Kpokiri et al. (2020) | *[map to S-Table 2 barrier categories]* | *[map to S-Table 2 facilitator categories]* | *[map to S-Table 2 strategy categories]* | *[enter key finding/outcome from extraction]* |
| 5 | Chukwu et al. (2024) | *[map to S-Table 2 barrier categories]* | *[map to S-Table 2 facilitator categories]* | *[map to S-Table 2 strategy categories]* | *[enter key finding/outcome from extraction]* |
| 6 | Akitan et al. (2024) | *[map to S-Table 2 barrier categories]* | *[map to S-Table 2 facilitator categories]* | *[map to S-Table 2 strategy categories]* | *[enter key finding/outcome from extraction]* |
| 7 | Abubakar & Tangiisuran (2020) | *[map to S-Table 2 barrier categories]* | *[map to S-Table 2 facilitator categories]* | *[map to S-Table 2 strategy categories]* | *[enter key finding/outcome from extraction]* |
| 8 | Kamere et al. (2022) | *[map to S-Table 2 barrier categories]* | *[map to S-Table 2 facilitator categories]* | *[map to S-Table 2 strategy categories]* | *[enter key finding/outcome from extraction]* |
| 9 | Aika et al. (2022) | *[map to S-Table 2 barrier categories]* | *[map to S-Table 2 facilitator categories]* | *[map to S-Table 2 strategy categories]* | *[enter key finding/outcome from extraction]* |
| 10 | Abubakar (2020) | *[map to S-Table 2 barrier categories]* | *[map to S-Table 2 facilitator categories]* | *[map to S-Table 2 strategy categories]* | *[enter key finding/outcome from extraction]* |

*Cells shaded amber are to be completed by the authors from the master data-extraction workbook; the manuscript reports only aggregate frequencies, so per-study attributions are not reproduced here to avoid misrepresenting the primary studies. When completed, the per-study marks must reconcile with the aggregate totals in main-text Table 2 (denominator = 10 studies): barriers 9, 8, 8, 7, 6, 6, 5, 4; facilitators 8, 7, 7, 6, 5, 5, 4, 3; strategies 9, 8, 8, 7, 6, 6, 5, 5, 4, 3.*
